# Impact of mixed plantation strategies on the nutrient concentrations of green and senescent leaves and their nutrient resorption efficiencies in temperate forests of the Loess Hilly Region

**DOI:** 10.3389/fpls.2025.1527930

**Published:** 2025-05-13

**Authors:** Senbao Lu, Yunming Chen, Jordi Sardans, Josep Peñuelas

**Affiliations:** ^1^ Pomology Institute, Shanxi Agricultural University, Taiyuan, Shanxi, China; ^2^ The Research Center of Soil and Water Conservation and Ecological Environment, Chinese Academy of Sciences and Ministry of Education, Yangling, Shaanxi, China; ^3^ Institute of Soil and Water Conservation, Chinese Academy of Sciences and Ministry of Water Resources, Yangling, Shaanxi, China; ^4^ University of Chinese Academy of Sciences, Beijing, China; ^5^ College of Soil and Water Conservation Science and Engineering, Northwest A&F University, Yangling, China; ^6^ CSIC, Global Ecology Unit CREAF-CSIC-UAB, Bellaterra, Catalonia, Spain; ^7^ CREAF, Cerdanyola del Vallès, Catalonia, Spain

**Keywords:** mixed species, leaf N and P concentrations, nutrient resorption efficiency, stand characteristics, soil properties

## Abstract

Mixed-species reforestation/afforestation has been a common practice for improving ecosystem nutrient sustainability. In the Loess Hilly Region, however, it remains unclear whether the mixture of dinitrogen (N_2_)-fixing species *Robinia pseudoacacia* with economic species *Amygdalus davidiana* and *Armeniaca sibirica* can improve nutrient concentrations of green and senescent leaves and their nutrient resorption. In 2022, we analyzed N and phosphorus (P) concentrations in green (GLNC, GLPC) and senescent leaves (SLNC, SLPC), resorption efficiencies (NRE and PRE), and relative resorption efficiency (RRE) in mixtures of *R. pseudoacacia* × *A. davidiana* and *R. pseudoacacia* × *A. sibirica* and monocultures of three species, considering tree structure and topsoil (0-20 cm) properties. One-way ANOVA followed by Tukey’s test showed that senescent leaves nutrient concentrations for *R. pseudoacacia* and Student’s t test that green and senescent leaves of *A. davidiana* and *A. sibirica* were significantly greater in mixed stands than monocultures. Based on the same statistical methods, NRE and PRE of *R. pseudoacacia* varied; those of *A. davidiana* and *A. sibirica* were significantly lower in mixed stands than monocultures. Additionally, RRE was < 100% for *R. pseudoacacia*, but > 100% for *A. davidiana* and *A. sibirica*; NRE and PRE was significantly positively associated in *A. sibirica* and all species. The correlation analysis demonstrated that crown area were significantly positively correlated with N and P concentrations of green and senescent leaves but negatively correlated with NRE and PRE for *A. davidiana* and *A. sibirica*, however, they were unrelated for *R. pseudoacacia* and all species. Further, a random forest model analysis showed that soil available P was main factor driving GLNC for individual rather than all species. Within each species, linear regression analysis revealed that NRE was significantly negatively correlated with SLNC; for *A. davidiana* and *A. sibirica*, NRE and PRE were significantly negatively correlated with SLNC and SLPC, respectively. In contrast, the PRE of all species was significantly negatively correlated with GLPC but positively correlated with SLPC. Overall, the results suggested that tree mixture increased N and P cycling more in *A. davidiana* and *A. sibirica* than in *R. pseudoacacia*, in the plant-soil system.

## 1 Introduction

Reforestation/afforestation has historically been a common practice used to control degraded land (Payn et al., 2015) and improve nutrient cycling in terrestrial ecosystems (Lamb et al., 2005). Although the global planted forest area increased by 123 million ha between 1990 and 2020 (FAO, 2020), plantations have not always promoted nutrient cycling (Delgado-Baquerizo et al., 2013; Jiang et al., 2019). This expansion often relies on monoculture plantations, which have led to declines in soil fertility, ecological services, and functional stability (Liu et al., 2018). In contrast, suitable combinations of mixed species have been demonstrated to increase ecosystem stability (Forrester et al., 2006) and enhance resilience to both biotic and abiotic stressors (Coll et al., 2018). In particular, planting dinitrogen (N_2_)-fixing species in mixed plantations can improve soil nitrogen availability and nutrient cycling (Forrester et al., 2006; Stewart et al., 2008; Nygren and Leblanc, 2015). However, current research has focused mainly on the mechanisms by which N_2_-fixing or non-N_2_-fixing species promote nutrient cycling in pure plantations (Cao and Chen, 2017; Deng et al., 2019; Xu et al., 2020). Therefore, how N_2_-fixing and non-N_2_-fixing species promote nutrient cycling differently in mixed versus pure plantations is unknown.

Compared with monocultures, interspecific competition for aboveground light and belowground nutrients consumes some of the energy used for nutrient uptake (Li et al., 2018; Zhu et al., 2020). This implies that mixed N_2_-fixing and non-N_2_-fixing species tend to maintain a balance of nutrient use strategies based on the environmental conditions (Jucker et al., 2015; Bu et al., 2019; Lin et al., 2022). For instance, leaf N and phosphorus (P) concentrations can be used to quantify the balance/status of bioelements between N_2_-fixing and non-N_2_-fixing species in mixed and monoculture plantations (Sardans et al., 2012; Peñuelas et al., 2013; Hayes et al., 2014; Sardans et al., 2021). Additionally, differences in the nitrogen (NRE) and phosphorus (PRE) resorption efficiencies of N_2_-fixing and non-N_2_-fixing species among mixed and pure plantations may reflect their adoption strategies to optimize nutrient use and conservation (Aerts, 1996; Reed et al., 2012) due to the influence of stand type on plant physiological activity (Chen and Chen, 2022). Therefore, nutrient interactions and balance among different species can be revealed by examining differences in leaf N and P concentrations and nutrient resorption efficiency (NuRE) of N_2_-fixing versus non-N_2_-fixing species in mixed plantations versus monocultures.

Leaf N and P concentrations have been widely reported to influence the N and P resorption efficiencies of mixed species (Chen et al., 2012; Vergutz et al., 2012; Deng et al., 2019). Previous studies have shown positive relationships between N and P resorption efficiencies and green leaf N and P concentrations (Zhang et al., 2018), but the relationships reported in other studies have been negative (Kobe et al., 2005; Vergutz et al., 2012; Liu et al., 2014) or not significant (Aerts, 1996; Sun et al., 2015). The difference is that N and P resorption efficiencies have negative effects on senescent leaf N and P concentrations because the decomposition of senescent leaves can increase the soil N and P concentrations (Wang et al., 2014; Zhou et al., 2016). These findings suggest that the difference in the N and P resorption efficiencies of mixed species can be reflected by the variation in green and senescent leaf N and P concentrations. Therefore, the relationship between the nutrient concentrations of green and senescent leaves and their resorption efficiencies can reveal the different roles of mixed N_2_-fixing and non-N_2_-fixing species in nutrient conservation.

Theoretically, leaf N and P concentrations and their resorption efficiencies in mixed and monoculture species are largely influenced by soil nutrient availability (Tully et al., 2013) through variations in stand characteristics (incl. tree structure and leaf morphological traits) and root distributions (Forrester et al., 2006; Jucker et al., 2015; Bu et al., 2019). Leaf N and P concentrations of mixed species are also indicated by leaf morphological traits in the variation of interspecific competition for light under different stand structures (Sanz-Perez et al., 2009; Duan et al., 2014; Metz et al., 2016). Furthermore, mixed N_2_-fixing species can increase soil N availability by rhizobia (Forrester et al., 2006; Paula et al., 2015; Waithaisong et al., 2020) but decrease soil P availability due to high P demand for N fixation (Augusto et al., 2013; Yuan et al., 2016). Thus, mixed species may modify N and P resorption efficiencies based on variations in soil N and P availability (Brant and Chen, 2015) and the potential effects of stand characteristics on leaf N and P concentrations (Vergutz et al., 2012; Liu et al., 2014; Xu et al., 2020). However, few studies have evaluated the effects of aboveground (stand) and belowground (soil nutrient availability) characteristics influenced by mixed planting strategies on leaf N and P concentrations and the resorption efficiencies of N_2_-fixing and non-N_2_-fixing species. Therefore, it is necessary to determine the feedback signals between leaf N and P concentrations and the nutrient resorption efficiencies of N_2_-fixing and non-N_2_-fixing species with soil N and P concentrations in monoculture and mixed plantations influenced by stand characteristics and soil nutrients to elucidate the mechanism by which mixed species affect nutrient cycling.

The historic degradation of natural vegetation across the Loess Plateau (covering 6.4 × 10^5^ km^2^) resulted in the occurrence of drought and soil erosion. In 1999, the Chinese government initiated the “Grain for Green” project with the objective of increasing plantation cover. By 2021, this initiative resulted in a 402.11% increase in plantation coverage (Meng et al., 2023). However, planted forests mostly consist of monocultures, which have a negative impact on the ecological environment of the Loess Plateau, including the depletion of soil fertility (Chen et al., 2015). The fast-growing N_2_-fixing tree species *Robinia pseudoacacia* L. has been widely introduced as a pioneer afforestation species into the Loess Plateau because of its high drought tolerance and resistance to low soil fertility (Cierjacks et al., 2013; Tsunekawa et al., 2014). However, the growth of *R. pseudoacacia* is limited by soil P availability and becomes increasingly limited by soil P as the stand ages (Cao and Chen, 2017; Liu et al., 2017). In addition, the branches that have withered over the long-term (19–25 years) growth of *R. pseudoacacia* plantations have led to concerns about their nutrient status. Although previous studies have examined the leaf N and P concentrations and N and P resorption efficiencies of mixed species (Bu et al., 2019; Lin et al., 2022), they have not included *R. pseudoacacia*, limiting our understanding of the nutrient status of mixed N_2_-fixing species (Forrester et al., 2006; Coll et al., 2018). Coincidentally, the native species *Amygdalus davidiana* (Carr.) C. de Vos ex Henry var. davidiana and *Armeniaca sibirica* (L.) Lam. for ecological (e.g., drought-resistant, well-developed roots and strong barberries) and economic benefits (e.g., the kernel of *A. davidiana* is often made for decoration, while the pulp of *A. sibirica* is edible) often mixed planted with *R. pseudoacacia* on the Loess Plateau. However, the mechanism by which the mixed species *R. pseudoacacia* alters leaf N and P concentrations and their resorption efficiencies by influencing stand and soil characteristics remain uncertain.

In the present study, green and senescent leaves of individual species and soil samples from *R. pseudoacacia* monocultures and mixed plantations were collected. The objectives of this study were to evaluate the effects of N_2_-fixing (*R. pseudoacacia*) and non-N_2_-fixing trees (*A. davidiana* and *A. sibirica*, respectively) in mixed and monoculture plantations on leaf N and P concentrations and their resorption efficiencies. These aims were achieved by comparing the differences in leaf N and P concentrations and resorption efficiencies between monocultures and mixed species and by examining the relationships between these variables and influencing factors such as stand characteristics and soil properties. It was hypothesized that (1) multispecies mixing could increase the leaf N and P concentrations of *R. pseudoacacia*, *A. davidiana* and *A. sibirica*; (2) species mixing could reduce the NRE but increase the PRE of *R. pseudoacacia*, *A. davidiana* and *A. sibirica* and increase the soil P limitation for *R. pseudoacacia*; (3) correlations between the leaf N concentration and NRE and the leaf P concentration and PRE could not be significant in *R. pseudoacacia* from different plantations but be significantly negative in *A. davidiana* and *A. sibirica.*

## 2 Materials and methods

### 2.1 Study site

The study was conducted in Ansai County, Shaanxi Province (36°35’21”-36°36’40” N, 109°13’12”-109°16’31” E), the hinterland of the Loess Plateau, where the mean annual temperature and precipitation are 8.8°C and 510 mm, respectively. The rainy season is concentrated between June and September (72.9%). The soils are categorized as Calcic Cambisols (FAO and UNESCO, 1974). Over the past two centuries, the original vegetation has been completely devastated. Secondary forest regeneration began naturally in the 1970s, and plantation efforts have increased significantly since 1999, following the project to return farmland to plantations. Due to soil erosion over a thousand years, the background data on soil properties are quite similar on the Loess Plateau. The initial afforestation densities varied across the the Loess Plateau depending on tree species and site conditions, and later, with the growth of vegetation, the stand density changed. Thus, after 24 years of growth (up to 2022), changes in interspecific relationships and individual differences of plantation vegetation due to stand density are a crucial part of the natural process of succession.

### 2.2 Experimental design

The investigation included five type of plantations on the Loess Hilly Region, and the stand density for each plot was determined through a counting process. There were categorized into two stand types: monoculture plantations of *R. pseudoacacia* (RP, 1083 ± 42 trees ha^-1^), *A. davidiana* (AD, 733 ± 33 trees ha^-1^), and *A. sibirica* (AS, 700 ± 58 trees ha^-1^) and mixed plantations of *R. pseudoacacia* (1192 ± 22 trees ha^-1^) with *A. davidiana* (325 ± 14 trees ha^-1^) (RPAD) and *R. pseudoacacia* (1025 ± 80 trees ha^-1^) with *A. sibirica* (308 ± 55 trees ha^-1^) (RPAS). Specifically, two tree species combinations were designed: RPAD, RP, and AD; and RPAS, RP, and AS. The objectives of this study were to determine which tree species presented greater leaf N and P concentrations and lower nutrient resorption efficiencies in mixed stands than in their corresponding monoculture stands and to identify the factors that influence these phenomena. The plantations were established approximately 16–25 years ago and are situated on loess soil with a southwest orientation. Three 20 m × 20 m plots from each plantation, 15 plots in total, separated by less than 3 km, and a 5-m buffer zone around each plot to avoid edge effects were selected for analysis (see **Supplementary Table S1**).

### 2.3 Vegetation survey and sampling

To ensure that the sample is representative, we select standard wood at the four corners and center of the quadrat. In each plantation stand, green and senescent leaves from pure plantations were collected from five representative trees of each species without withered branches, and samples from mixed plantations were taken from adjacent two different species. The diameter at breast height (DBH, cm) was measured using a tree diameter tape. The average tree height (AH, m) was determined using a height gauge. Meanwhile, the crown area (CA, m^2^) was determined using a tapeline. When the biomass of plants was at its peak (in mid-August 2022), intact and complete green leaves were collected from the lower, middle, and upper canopies using high branch shears. From November to December 2022, fallen senescent leaves were collected from nylon nets (1 m × 1 m, 0.1 mm) suspended below the canopy. Based on the characteristics of defoliation season of different tree species, the collection time of senescent leaves of *R. pseudoacacia* and *A. davidiana* was set in late December 2022, while that of *A. sibirica* was set in mid-September 2022. Intact 5-10 green leaf samples without pests and diseases were selected and stored in ice bags to measure morphological features such as specific leaf area (SLA), leaf dry matter content (LDMC), and leaf tissue density (LD). Other green and senescent leaves were oven-dried to a constant mass at 105°C for 10 min and at 70°C for 24 h to determine the green leaf nitrogen (GLNC) and phosphorus (GLPC) concentrations and the senescent leaf nitrogen (SLNC) and phosphorus (SLPC) concentrations. The stand characteristics of the monocultures and mixed stands are shown in **Supplementary Table S2**.

Soil samples were collected from 0–20 cm in an S shape with five loci per plot using a 4 cm auger under the representative trees of each plantation, in mid-August 2022. Before the soil passed through the 2 mm mesh screen, we manually removed the roots, stones, and debris. The soil was divided into two parts: one part was air-dried, ground, and passed through a 0.15 mm sieve to determine the soil chemical properties, e.g., soil organic carbon (SOC), total N (TN), total P (TP), available N (AN), and available P (AP) concentrations; the other part was shade-dried and passed through a 1 mm porous sieve to determine the pH and particle composition (i.e., sand content, SA; silt content, SI; clay content, CL). At a depth of 0–20 cm, undisturbed soil was collected from each plot using a ring knife (100 cm^3^) to determine the bulk density (BD).

### 2.4 Sample measurement

The N concentrations of green (GLNC, g kg^-1^) and senescent (SLNC, g kg^-1^) leaves were determined by the Kjeldahl method (Kjeltec 2300 analyzer unit, Foss Tecator, Hoganas, Sweden) after digestion in H_2_SO_4_–H_2_O_2_ solution. The P concentrations of green (GLPC, g kg^-1^) and senescent (SLPC, g kg^-1^) leaves were determined by the molybdenum yellow method (U-2800 spectrophotometer, Shanghai, China). Specific leaf area (SLA, cm^2^ g^-1^), leaf dry matter content (LDMC, g g^-1^) and leaf tissue density (LD, g cm^-3^) were calculated using the following equation:


(1) $$\text{SLA}=\frac{\text{LA}}{\text{DW}}$$

(2) $$\text{LDMC}=\frac{\text{DW}}{\text{FW}}$$

(3) $$\text{LD}=\frac{\text{DW}}{\text{LT}\times \text{LA}}$$

where leaf fresh weight (FW) and dry weight (DW) were determined using an electronic balance (precision 0.001 g), leaf thickness (LT, mm) was determined using a digital vernier caliper, and leaf area (LA, cm^2^) was determined using an Epson Perfection V850 Pro scanner (Epson (China) Co., Ltd. Beijing, China) and ImageJ software.

The soil organic C (SOC, g kg^-1^) concentration was determined by the calorific boiling and combustion with the H_2_SO_4_-K_2_Cr_2_O_7_ method (Nelson and Sommers, 1982). The soil total N (TN, g kg^-1^) concentration was determined by the Kjeldahl method (Bremner and Mulvaney, 1982), whereas the available N (AN, mg kg^-1^) concentration was measured by alkaline hydrolysis diffusion (Bao, 2000). The soil total P (TP, g kg^-1^) and available P (AP, mg kg^-1^) concentrations were determined by the molybdenum blue method after extraction with H_2_SO_4_–HClO_4_ and NaHCO_3_, respectively (Murphy and Riley, 1962). The soil pH was measured using an automatic acid–base titrator (PB-10 standard pH meter; Sartorius, Göttingen, Germany) with a water/soil ratio of 2.5:1. The cutting ring method was used to determine the soil BD (g cm^-3^) (Bao, 2000). The soil particle composition (SA/SI/CL, %) was determined using a Mastersizer 2000 Laser Particle Size Analyzer (Malvern Panalytical, Malvern, UK). The soil physicochemical properties are listed in **Supplementary Table S3**.

### 2.5 Data analysis

#### 2.5.1 Nutrient resorption efficiency

The nutrient concentration percentage changes in green leaves were calculated using the following equation:


(4) $${\Delta \text{NuCgreen}}_{\text{mix}-\text{mono}}=\frac{{\text{NuCgreen}}_{\text{mix}}-{\text{NuCgreen}}_{\text{mono}}}{{\text{NuCgreen}}_{\text{mono}}}\times 100$$

where ΔNuCgreen_mix−mono_ (%) is the percentage changes in green leaf nitrogen (N) or phosphorus (P) concentrations between mixed and monoculture stands (see **Supplementary Table S4**), with NuCgreen_mix_ and NuCgreen_mix_ being the N or P concentrations in mixed and monoculture stands, respectively.

The nutrient concentration percentage changes in senescent leaves were calculated using the following equation:


(5) $${\Delta \text{NuCsenescent}}_{\text{mix}-\text{mono}}=\frac{{\text{NuCsenescent}}_{\text{mix}}-{\text{NuCsenescent}}_{\text{mono}}}{{\text{NuCsenescent}}_{\text{mono}}}\times 100$$

where ΔNuCsenescent_mix-mono_ (%) is the percentage changes in senescent leaf N or P concentrations between mixed and monocultures stands (see **Supplementary Table S4**), with NuCsenescent_mix_ and NuCsenescent_mix_ being the N or P concentrations in mixed and monoculture stands, respectively.

#### 2.5.2 Nutrient resorption efficiency, relative nutrient resorption efficiency, and associated percentage changes

**Figure 1:**
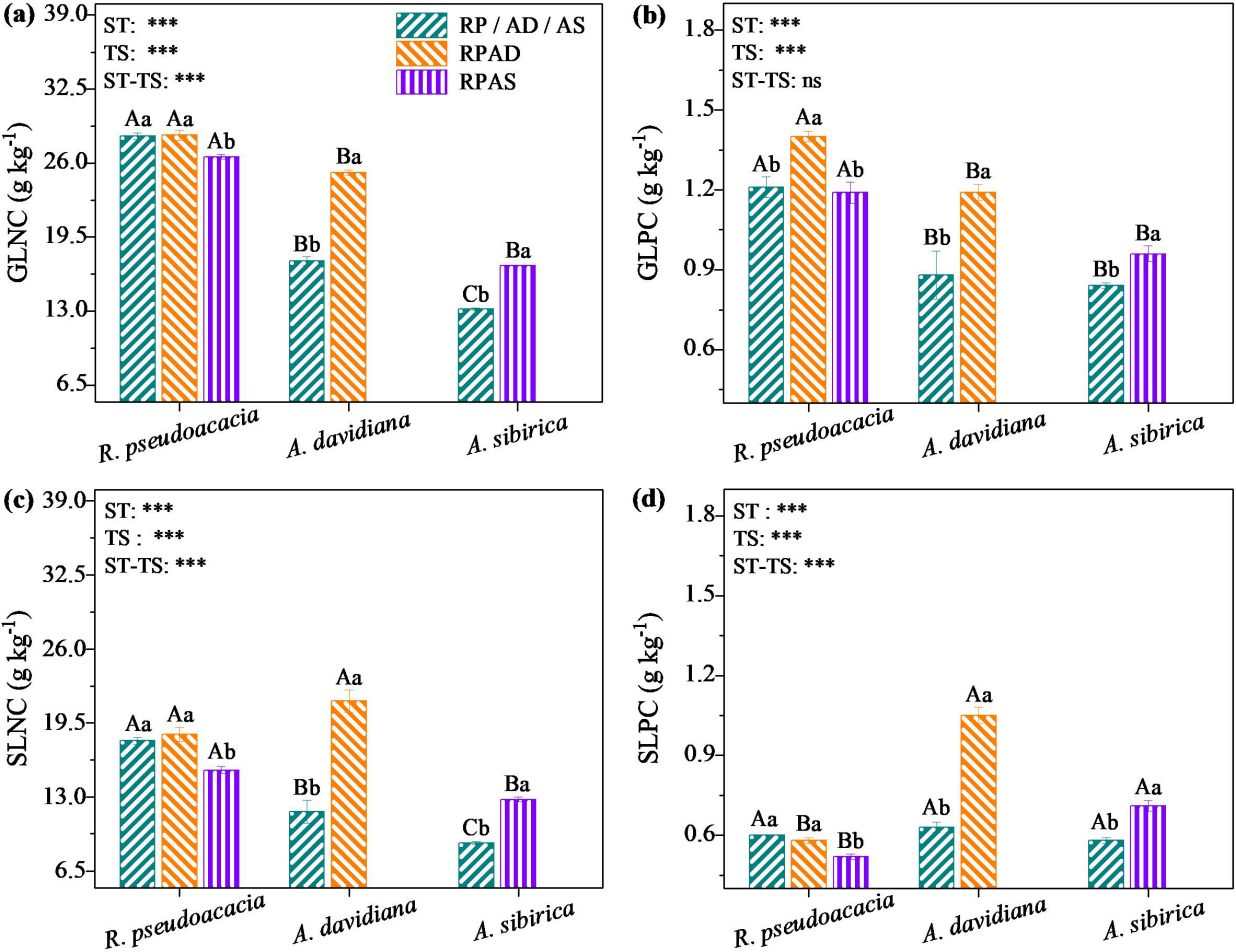
Changes in green leaf nitrogen (GLNC, **a**) and phosphorus (GLPC, **b**) concentrations, and senescent leaf nitrogen (SLNC, **c**) and phosphorus (SLPC, **d**) concentrations of different tree species in monocultures and mixed stands. The results of the two-way ANOVA for stand type (ST) and tree species (TS) and their interaction (ST-ST) on green and senescent leaf nitrogen and phosphorus concentrations are shown in the figures. Different uppercase letters indicate significant differences among different tree species for the same stand type, whereas different lowercase letters above the bars indicate significant differences among the same tree species for different stand types (***, *P* < 0.001; ns, *P* > 0.05).

Nutrient resorption efficiency (NuRE, %) in monoculture stands was calculated using the following equation:


(6) $${\text{NuRE}}_{\text{mono}}=(1-\frac{{\text{NuCsenescent}}_{\text{mono}}}{{\text{NuCgreen}}_{\text{mono}}}\times \text{MLCF})\times 100$$

where NuRE_mono_ is the N or P resorption efficiency in monoculture stands (**Figure 1a**), with NuCsenescent_mono_ and NuCgreen_mono_ being the N or P concentrations in respective senescent and green leaves, and MLCF is the angiosperm-specific mass loss correction factor (0.784; Vergutz et al., 2012).

Nutrient resorption efficiency in mixed stands was calculated using the following equation:


(7) $${\text{NuRE}}_{\text{mix}}=(1-\frac{{\text{NuCsenescent}}_{\text{mix}}}{{\text{NuCgreen}}_{\text{mix}}}\times \text{MLCF})\times 100$$

where NuRE_mix_ is the N or P resorption efficiency in mixed stands (**Figure 1b**), with NuCsenescent_mix_ and NuCgreen_mix_ being the N or P concentrations in senescent and green leaves, respectively.

**Figure 2:**
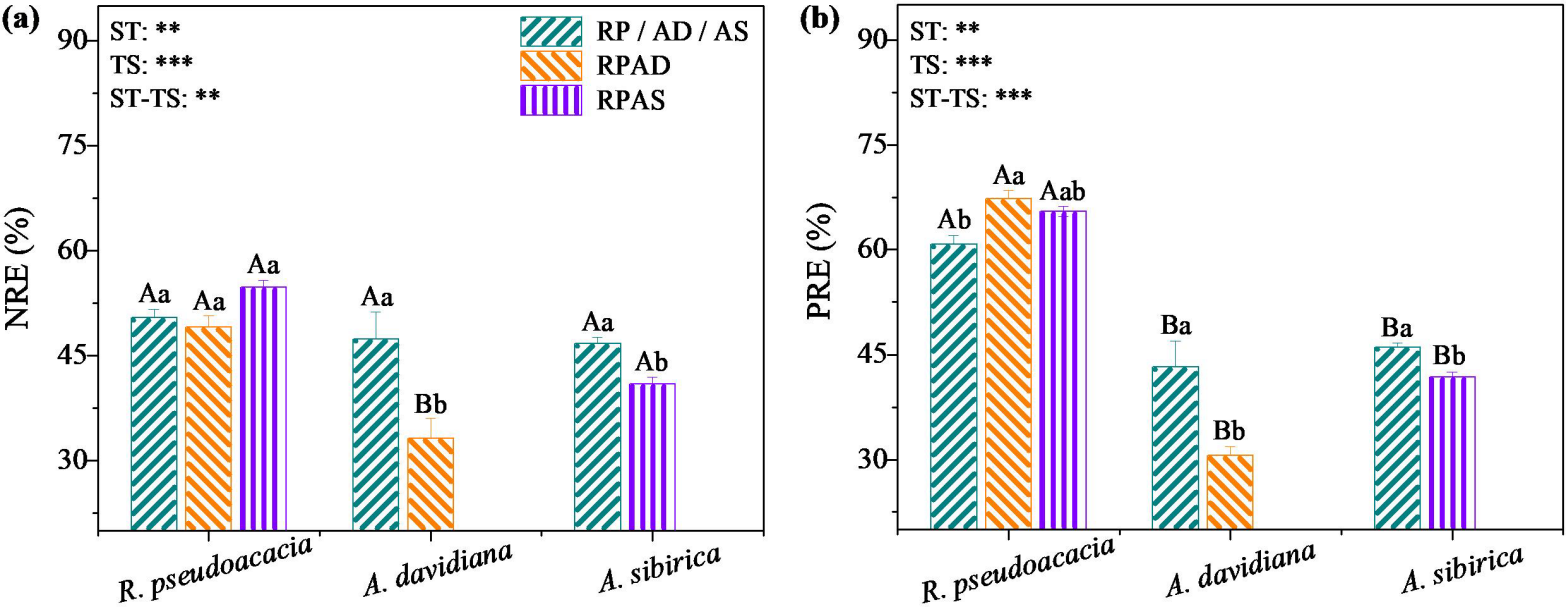
Changes in nitrogen (NRE, **a**) and phosphorus (PRE, **b**) resorption efficiencies of different tree species in monoculture and mixed stands. Results of the two-way ANOVA for stand type (ST) and tree species (TS) and their interaction (ST-ST) on green and senescent leaf nitrogen and phosphorus concentrations are shown in the figures. Different uppercase letters indicate significant differences among different tree species for the same plantation type, whereas different lowercase letters above the bars indicate significant differences among the same tree species for different plantation types (***, *P* < 0.001; **, *P* < 0.01).

Relative nutrient resorption efficiency (RRE, %) in monoculture/mixed stands was calculated using the following equation:


(8) $${\text{RRE}}_{\text{mono}/\text{mix}}=\frac{{\text{NRE}}_{\text{mono}/\text{mix}}}{{\text{PRE}}_{\text{mono}/\text{mix}}}\times 100$$

where RRE_mono/mix_ is the relative nutrient resorption efficiency in monoculture/mixed stands (**Figure 2a**), with NRE_mono/mix_ and PRE_mono/mix_ being the respective N and P resorption efficiency in momoculture/mixed stands. When RRE_mono/mix_ > 100%, N limits plant growth; when RRE_mono/mix_ < 100%, P limits plant growth; and when RRE_mono/mix_ = 100%, N and P have the same limitations on plant growth (Reed et al., 2012).

The percentage changes in nutrient resorption efficiency were calculated using the following equation:


(9) $${\Delta \text{NuRE}}_{\text{mix}-\text{mono}}=\frac{{\text{NuRE}}_{\text{mix}}-{\text{NuRE}}_{\text{mono}}}{{\text{NuRE}}_{\text{mono}}}\times 100$$

where ΔNuRE_mix-mono_ (%) is the percentage changes in N or P resorption efficiency between mixed and monoculture stands (see **Supplementary Table S4**), with NuRE_mix_ and NuRE_mono_ being the leaf N or P resorption efficiency in mixed stands and monocultures, respectively.

### 2.6 Statistical analysis

Data were tested for normality and homogeneity using the Shapiro–Wilk and Levene tests. When necessary, the data were log transformed. One way analysis of variance (ANOVA) followed by Tukey’s test was performed using SPSS 23.0 (SPSS Inc., Chicago, Illinois, USA) to determine the differences in green and senescent leaves concentrations, nutrient resorption, and relative nutrient resorption of *R. pseudoacacia* among the different stands and the differences among *R. pseudoacacia*, *A. davidiana*, and *A. sibirica* monocultures. Student’s t test was performed using SPSS software to examine differences between monoculture and mixed stands in green and senescent leaves concentrations, nutrient resorption, and its ratio of *A. davidiana*/*A. sibirica*, and examine differences between *R. pseudoacacia and A. davidiana* with *A. sibirica* in the same mixed stand. For further analysis, two-way ANOVA was performed using SPSS software to determine the effects of stand type, tree species, and their interaction on leaf nutrient concentrations and resorption efficiency. Linear regression analysis was performed using Origin Pro 2024b (OriginLab, Northampton, Massachusetts, USA) to determine the relationships between leaf nutrient concentrations, resorption efficiencies, and soil nutrient concentrations and between leaf nutrient concentrations and resorption efficiencies of specific and all species in monocultures and mixed stands. Pearson correlation analysis was performed using Origin Lab 2024b with “Correlation Plot” applications to confirm the correlations among the leaf nutrient concentrations, resorption efficiencies, and influencing factors (stand characteristics and soil properties) of the individuals and all species in the different stands. After standardizing the data with a Z-Score, random forest model analysis was performed using the “randomForest” package in R 4.2.2 (R Core Team, 2022) to determine the influencing factors that contribute to the leaf nutrient concentrations and resorption efficiencies of individuals and all species in pure and mixed stands. The significance level of the statistical analysis was set at *P* < 0.05.

## 3 Results

### 3.1 Leaf nutrient concentrations

In our study, two-way ANOVA revealed the significant effects of stand type, tree species, and their interaction on leaf nutrient concentrations (*P* < 0.05, **Figure 1**). Specifically, GLNC of *R. pseudoacacia* in the RPAS stand was 6.41% lower (*P* < 0.05; **Figure 1a**; **Supplementary Table S4**) and the GLPC of *R. pseudoacacia* in the RPAD stand was 15.70% higher than those in the RP stand (*P* < 0.05; **Figure 1b**; **Supplementary Table S4**). Additionally, the GLNC and GLPC of *A. davidiana* in the RPAD stand were 44.48% and 35.23% higher (*P* < 0.05; **Figures 1a, b**; **Supplementary Table S4**), respectively, and those of *A. sibirica* in the RPAS stand were 28.83% and 14.29% higher than those of their respective monocultures (*P* < 0.05; **Figures 1a, b**; **Supplementary Table S4**).

Furthermore, the SLNC and SLPC of *R. pseudoacacia* in the RPAS stand were 14.44% and 13.33% lower than those in the RP stand, respectively (*P* < 0.05; **Figures 1c, d**; **Supplementary Table S4**). Moreover, the SLNC and SLPC of *A. davidiana* in the RPAD stand were 83.08% and 66.67% greater (*P* < 0.05; **Figures 1c, d**; **Supplementary Table S4**), respectively, and those of *A. sibirica* in the RPAS stand were 42.75% and 22.41% greater than those in the corresponding monoculture stands (*P* < 0.05; **Figures 1c, d**; **Supplementary Table S4**).

### 3.2 Leaf nutrient resorption efficiency and its relationship

Two-way ANOVA revealed the significant effects of stand type, tree species, and their interaction on NRE (Equation 6) and PRE (Equation 7) (*P* < 0.05, **Figure 2**). Compared with that in the RP stand, the PRE of *R. pseudoacacia* in RPAD stand was 10.73% greater (*P* < 0.05; **Figure 2b**; **Supplementary Table S4**). The NRE and PRE of *A. davidiana* in the RPAD stand were 29.93% and 29.35% lower than those in the AD stand, respectively, and those of *A. sibirica* in the RPAS stand were 12.21% and 9.31% lower than those in the AS stand, respectively (*P* < 0.05; **Figures 2a, b**; **Supplementary Table S4**).

**Figure 3:**
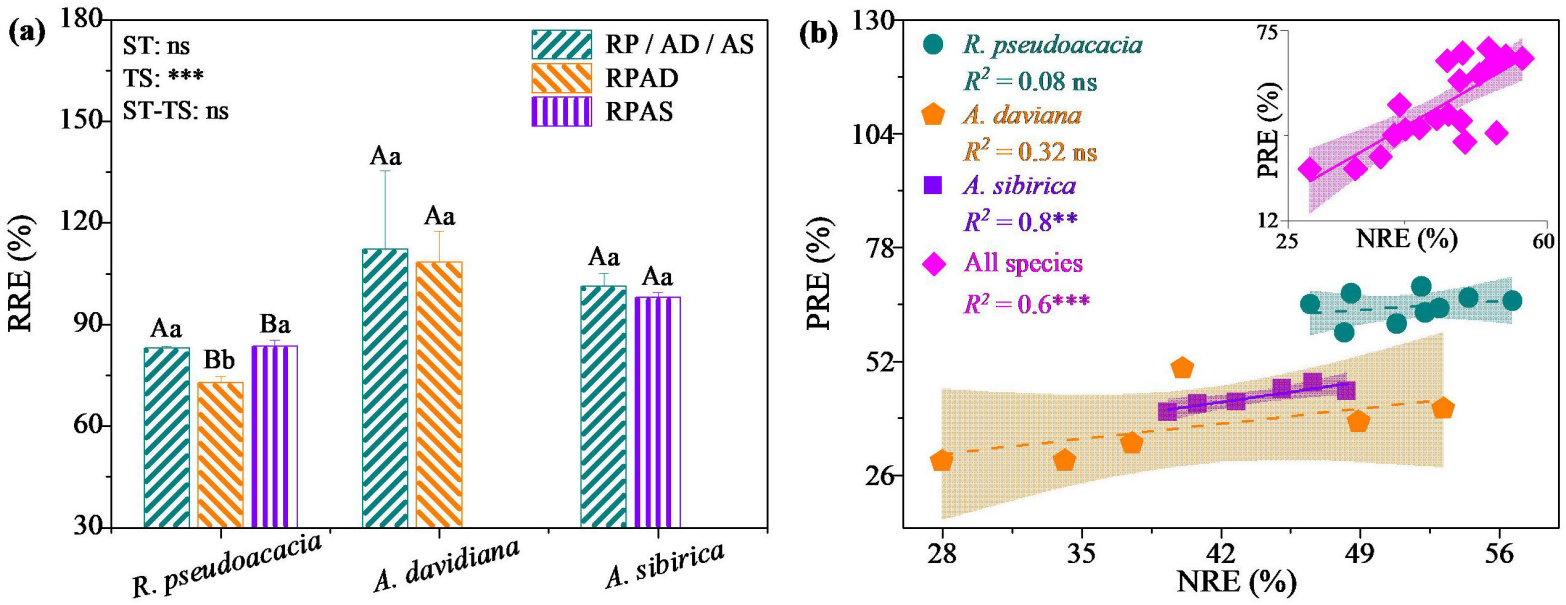
The relative resorption efficiency (RRE) of different tree species in monocultures and mixed stands **(a)** and the linear regression of associations between nitrogen (NRE) and phosphorous (PRE) resorption efficiencies of individuals and all species in monocultures and mixed stands **(b)**. The results of the two-way ANOVA for stand type (ST) and tree species (TS) and their interaction (ST-ST) on green and senescent leaf nitrogen and phosphorus concentrations are shown in the figures. Different uppercase letters indicate significant differences among different tree species for the same stand type, while different lowercase letters above the bars indicate significant differences among the same tree species for different stand types (***, *P* < 0.001; **, *P* < 0.01; ns, *P* > 0.05).

Two-way ANOVA revealed the significant effects of stand type, tree species, and their interaction on relative nutrient efficiency (RRE, Equation 8) (*P* < 0.05, **Figure 3a**). Furthermore, the RRE of *R. pseudoacacia* was less than 100% and 12.05% lower in the RPAD stand than in the RP stand (*P* < 0.05), but those of *A. davidiana and A. sibirica* were greater than 100% and did not differ significantly between the pure and mixed plantations (*P* > 0.05) (**Figure 3a**; **Supplementary Table S4**). Moreover, linear regression analysis revealed that the NREs of *A. sibirica* and all species in the different stands were significantly positively correlated with PRE (*P* < 0.05, **Figure 3b**).

### 3.3 Relationships between leaf nutrient concentrations, nutrient resorption efficiencies, and influencing factors

**Figure 4:**
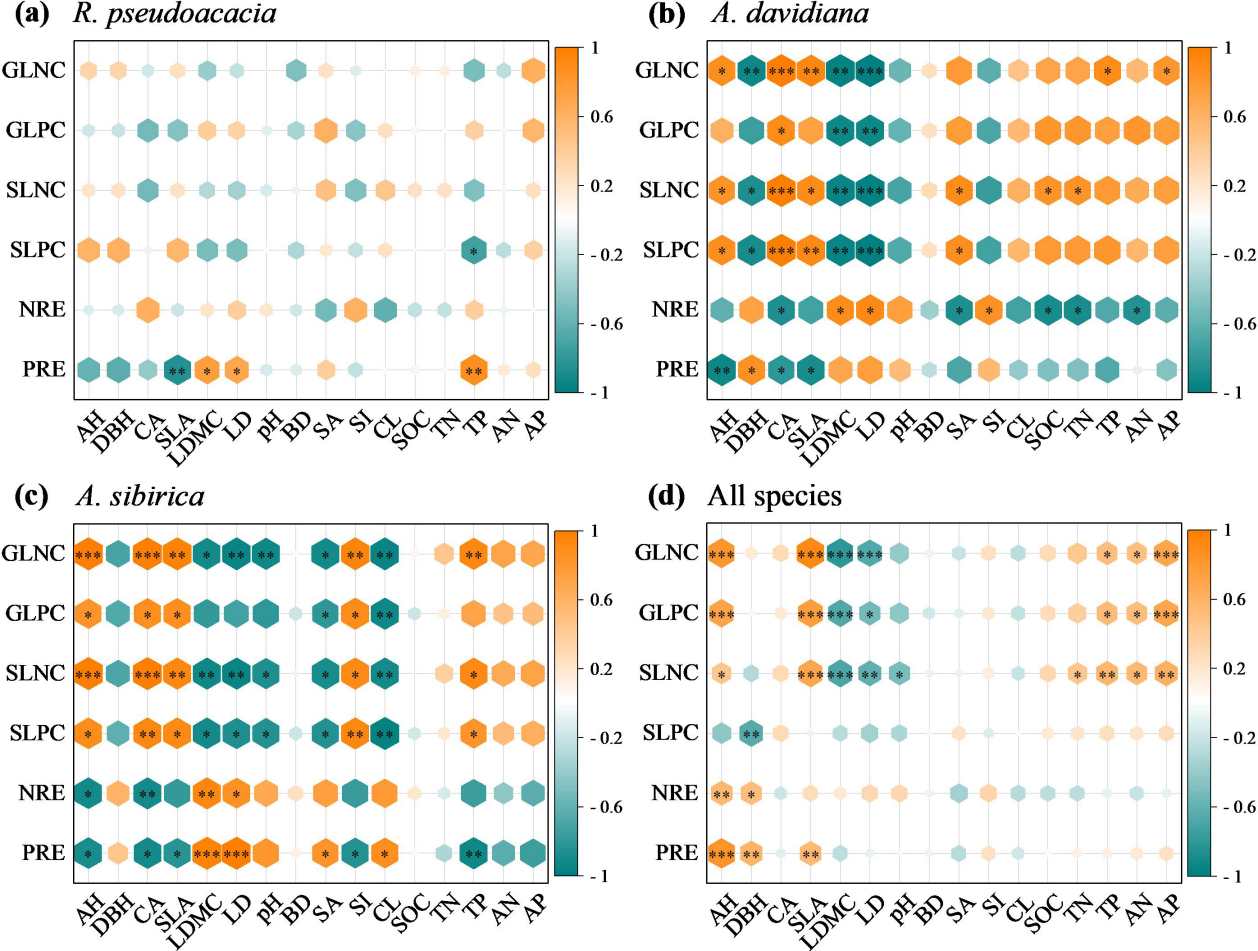
Pearson coefficients of the correlations between leaf nutrient concentrations, resorption efficiencies and stand characteristics and the soil properties of *R. pseudoacacia* **(a)**, *A. davidiana* **(b)**, *A. sibirica* **(c)**, and all species **(d)** in monoculture and mixed stands. GLNC, green leaf nitrogen concentration; GLPC, green leaf phosphorus concentration; SLNC, senescent leaf nitrogen concentration; SLPC, senescent leaf phosphorus concentration; NRE, nitrogen resorption efficiency; PRE, phosphorus resorption efficiency; AH, average tree height; DBH, diameter at breast height; CA, crown area; SLA, specific leaf area; LDMC, leaf dry matter content; LD, leaf tissue density; pH, soil pH; BD, soil bulk density; SA, soil sand content; SI, soil silt content; CL, soil clay content; SOC, soil organic carbon concentration; TN, soil total nitrogen concentration; TP, soil total phosphorus concentration; AN, soil available nitrogen concentration; AP, soil available phosphorus concentration. ***, *P* < 0.001; **, *P* < 0.01; *, *P* < 0.05.

Pearson’s correlation coefficient was used to determine the relationships between the leaf nutrient concentration, resorption efficiency, and stand characteristics and soil physicochemical properties of individuals and all species (**Figure 4**). For individual species, the SLPC of *R. pseudoacacia* was significantly negatively correlated with the soil total phosphorus concentration; PRE was significantly positively correlated with the leaf dry matter content, leaf tissue density, and soil total phosphorous concentration but significantly negatively correlated with the specific leaf area (*P* < 0.05, **Figure 4a**). The GLNC, GLPC, SLNC, and SLPC of *A. davidiana* and *A. sibirica* were significantly positively correlated with crown area (*P* < 0.05, **Figures 4b, c**), but the opposite was true for NRE and PRE (*P* < 0.05, **Figures 4b, c**). In all species, GLNC, SLNC, and SLPC were significantly positively correlated with average tree height, soil total phosphorus concentration, and soil available nitrogen and phosphorus concentrations. The SLPC was significantly negatively correlated with diameter at breast height. The NRE and PRE were significantly positively correlated with average tree height and diameter at breast height (*P* < 0.05, **Figure 4d**).

### 3.4 Factors contributing to leaf nutrient concentrations and nutrient resorption efficiencies

**Figure 5:**
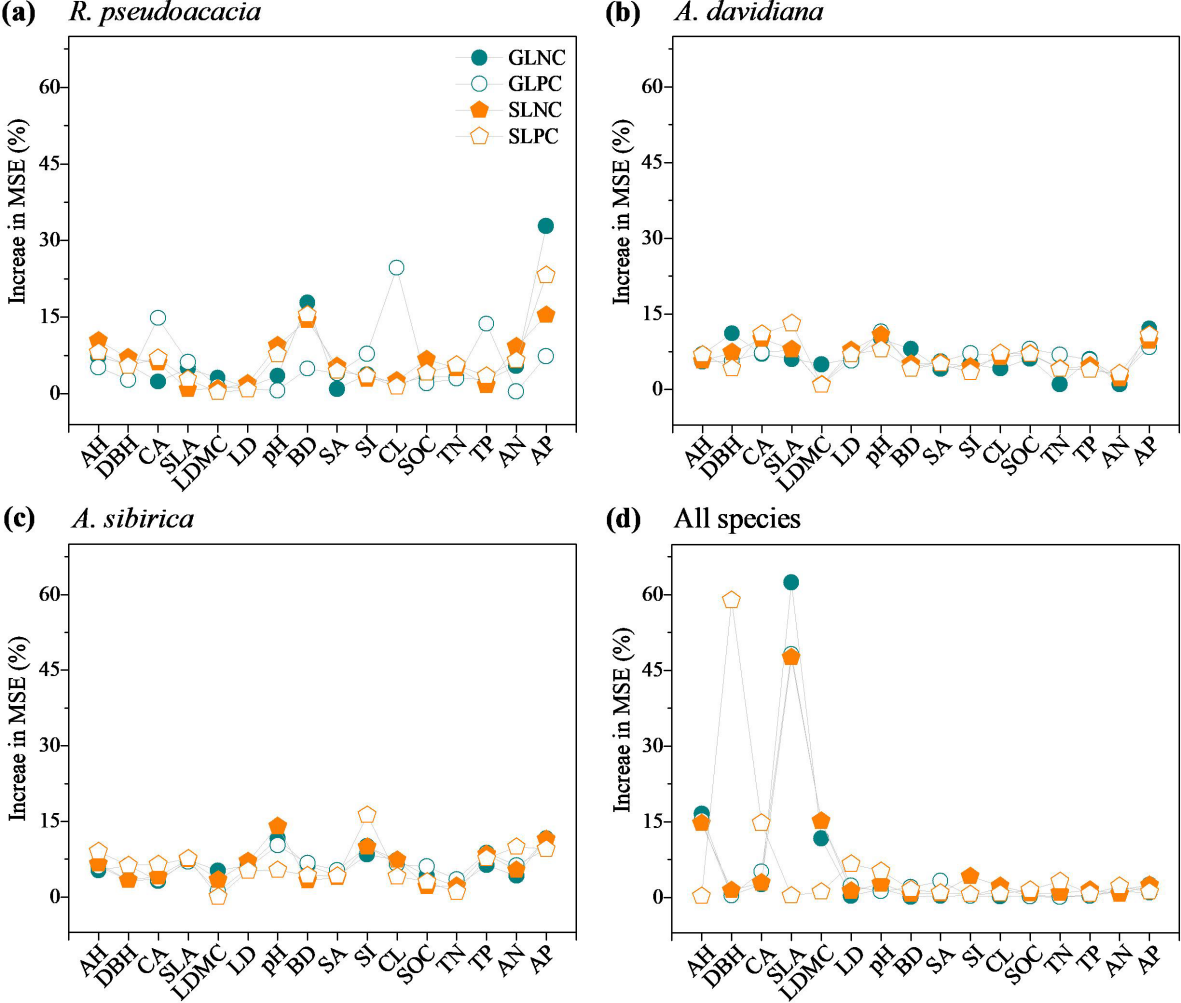
Random forest model analysis of the percentage increases in the mean square error (MSE, %) of variables in monocultures and mixed stands contributing to the changes in leaf nutrient concentrations of *R. pseudoacacia* **(a)**, *A. davidiana* **(b)**, *A. sibirica* **(c)**, and all species **(d)**. GLNC, green leaf nitrogen concentration; GLPC, green leaf phosphorus concentration; SLNC, senescent leaf nitrogen concentration; SLPC, senescent leaf phosphorus concentration; AH, average tree height; DBH, diameter at breast height; CA, crown area; SLA, specific leaf area; LDMC, leaf dry matter content; LD, leaf tissue density; pH, soil pH; BD, soil bulk density; SA, soil sand content; SI, soil silt content; CL, soil clay content; SOC, soil organic carbon concentration; TN, soil total nitrogen concentration; TP, soil total phosphorus concentration; AN, soil available nitrogen concentration; AP, soil available phosphorus concentration.

**Figure 6:**
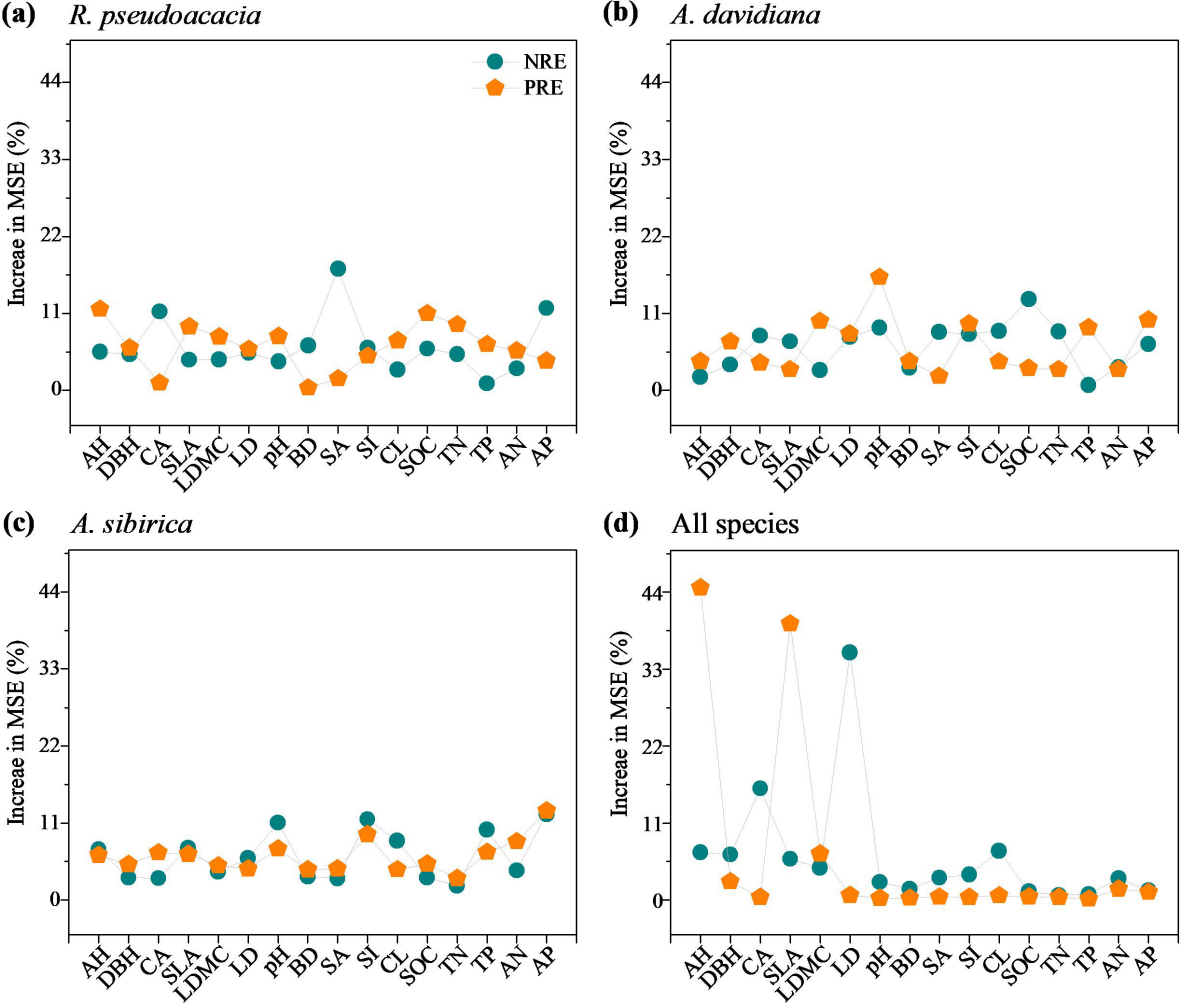
Random forest model analysis of the percentage increases in the mean square error (MSE, %) of variables in monocultures and mixed stands contributing to the changes in leaf nutrient resorption efficiencies of *R. pseudoacacia* **(a)**, *A. davidiana* **(b)**, *A. sibirica* **(c)**, and all species **(d)**. NRE, nitrogen resorption efficiency; PRE, phosphorus resorption efficiency; AH, average tree height; DBH, diameter at breast height; CA, crown area; SLA, specific leaf area; LDMC, leaf dry matter content; LD, leaf tissue density; pH, soil pH; BD, soil bulk density; SA, soil sand content; SI, soil silt content; CL, soil clay content; SOC, soil organic carbon concentration; TN, soil total nitrogen concentration; TP, soil total phosphorus concentration; AN, soil available nitrogen concentration; AP, soil available phosphorus concentration.

A random forest model was used to determine the relative importance of influencing factors to leaf nutrient concentrations and their resorption efficiencies for individuals and all species (**Figures 5**, **6**). Among the individual species, soil available P concentration had large effects on the GLNC of *R. pseudoacacia* (32.84%), *A. davidiana* (12.07%), and *A. sibirica* (11.43%) (**Figures 5a–c**). The clay content had large effects on the GLPC of *R. pseudoacacia* (24.67%), and pH had effects on *A. davidiana* (11.54%), and *A. sibirica* (10.25%). The soil available P concentration had strong effects on the SLNC of *R. pseudoacacia* (15.44%), and pH had effects on *A. davidiana* (10.87%), and *A. sibirica* (14.01%). The soil available P concentration had large effects on the SLPC of *R. pseudoacacia* (23.22%), *A. davidiana* (10.87%), and *A. sibirica* (9.53%) (**Figures 5a–c**). For all species, specific leaf area had strong effects on the GLNC (62.42%), GLPC (47.6%), and SLNC (47.6%), and diameter at breast height on the SLPC (58.93%) (**Figure 5d**).

For individual species, soil sand content (17.36%) had large effects on the NRE of *R. pseudoacacia*, soil organic C concentration (13.1%) had effects on the NRE of *A. davidiana*, soil available P concentration (12.73%) had effects on the NRE of *A. sibirica* (**Figures 6a–c**). The average tree height (11.62%) had strong effects on the NRE of *R. pseudoacacia*, soil pH (16.15%) had great effects on the NRE of *A. davidiana*, soil available P (12.73%) concentrations had effects on NRE of *A. sibirica.* For all species, leaf dry matter content (35.34%) effected the NRE, and average tree height (44.64%) had effects on the PRE (**Figure 6d**).

### 3.5 Relationships between leaf nutrient concentrations and nutrient resorption efficiencies

**Figure 7:**
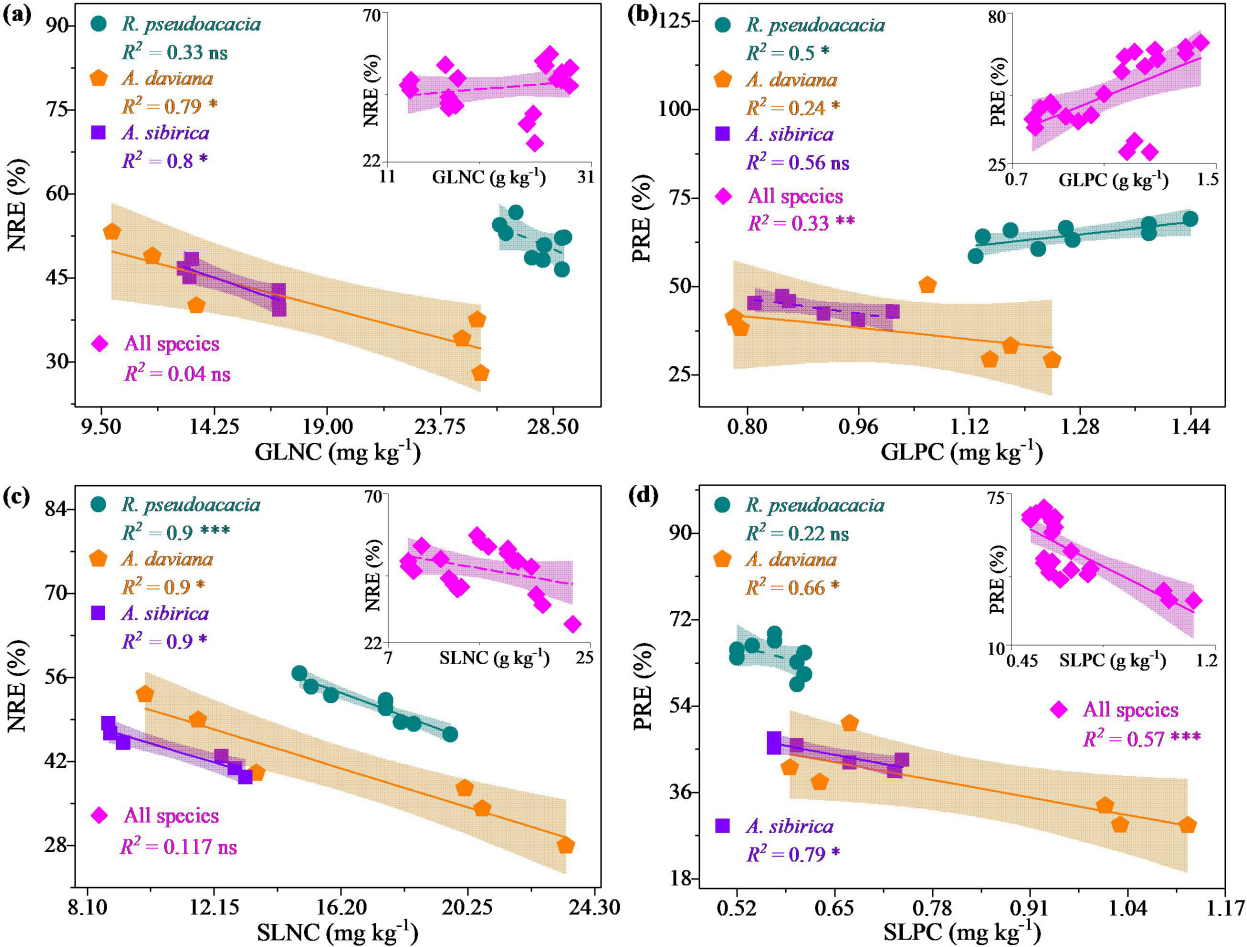
Linear regression analysis of the associations between nitrogen resorption efficiency (NRE) and green leaf nitrogen concentration (GLNC) **(a)**, phosphorus resorption efficiency (PRE) and green leaf phosphorus concentration (GLPC) **(b)**, NRE and senescent leaf nitrogen concentration (GLNC) **(c)**, and PRE and senescent leaf phosphorus concentration (SLPC) **(d)** of specific and all species in monoculture and mixed stands. ***, *P* < 0.001; **, *P* < 0.01; *, *P* < 0.05; ns, *P* > 0.05.

Linear regression analysis revealed different correlations between leaf nutrient concentrations and nutrient resorption across individuals and all species (**Figure 7**). For specific species, the PRE of *R. pseudoacacia* was significantly positively correlated with the GLNC (*P* < 0.05, **Figure 7b**), and the opposite was true between the NRE and SLNC (*P* < 0.05, **Figure 7c**). The NREs of *A. davidiana* and *A. sibirica* were significantly negatively correlated with GLNC and SLNC (*P* < 0.05; **Figures 7a, c**) and were the same between PRE and SLPC (*P* < 0.05, **Figure 7d**). For all species, PRE was significantly positively correlated with GLPC (*P* < 0.05, **Figure 7b**) and significantly negatively correlated with SLNC (*P* < 0.001, **Figure 7d**).

## 4 Discussion

### 4.1 Leaf nutrient concentrations and their influencing factors

Previous studies have reported that N_2_-fixing species promoted the expansion of root networks and that mycorrhizal systems increase rhizobial activity, thereby increasing the concentration of soil available N (Forrester et al., 2006; Nygren and Leblanc, 2015; Yuan et al., 2016). Additionally, the decomposition of senescent leaves of *R. pseudoacacia* can further increase the N resources of *A. davidiana* and *A. sibirica* (Forrester et al., 2006; Voigtlaender et al., 2012; Paula et al., 2015). Thus, the soil total N concentration was higher in the mixed stands than in the monoculture stands (**Supplementary Table S3**). However, the GLNC and specific leaf area were not significantly positively correlated for *R. pseudoacacia* but were significantly positively correlated for *A. davidiana* and *A. sibirica* (**Figures 4a–c**), partially supporting Hypothesis 1. One reasonable explanation is that the canopy of mixed *R. pseudoacacia* is located above *A. davidiana* and *A. sibirica*, the decrease in light intensity is lower by the mixed *R. pseudoacacia* but higher for mixed *A. davidiana* and *A. sibirica* compared to mono species. Particularly, in the mixed stand, the light intensity of *R. pseudoacacia* was less affected by *A. davidiana*, while that of which was more affected by *A. sibirica*. Thus, the GLNC of *R. pseudoacacia* did not differ between the RPAD and RP stands, whereas lower in the RPAS than in the RP stand (**Figure 1a**), contrary to Hypothesis 1. These findings indicate that the GLNC of *R. pseudoacacia* is not only affected by the total N content of soil but also by the specific leaf area (Sanz-Perez et al., 2009; Liu et al., 2014; Zhu et al., 2020).

The GLPC of *R. pseudoacacia* was greater in the RPAD stand than in the RP stand, but there was no difference between the RPAS and RP stands (**Figure 1b**; **Supplementary Table S4**), which was partially consistent with Hypothesis 1. This could be involved stand characteristics and soil properties interact to affect the GLPC values of *R. pseudoacacia* in monoculture and mixed stands (Xu et al., 2020; Sardans et al., 2021). For instance, crown area, clay content, soil total P concentration were not significantly correlated with the GLPC values of *R. pseudoacacia* (**Figure 4a**), but they had great relative contributions to the GLPC of *R. pseudoacacia* (**Figure 5a**). Consistent with the fact that the GLPC values of *A. davidiana* and *A. sibirica* in mixed stands were significantly positively correlated with crown area (**Figures 4b, c**), the GLPC and crown area values of which were greater in the mixed plantations than in the monocultures (**Figure 1b**; **Supplementary Table S4**). It can be inferred that understory trees tend to allocate more P to increase leaf metabolic activity. Additionally, low soil sand content resulted in high soil porosity and water storage (Sardans et al., 2011; Metz et al., 2016), beneficial for root expansion and microbial activity (Nygren and Leblanc, 2015; Ma et al., 2017) and indirectly improves the rapid release of P from organic matter (Blank and Morgan, 2014; Coll et al., 2018; Jiang et al., 2019; Waithaisong et al., 2020). As a result, the soil sand content was lower in the RPAS stand than in the AS stand (**Supplementary Table S3**).

Decomposing senescent leaves play an important role in nutrient cycling in the plant–soil ecosystem. In particular, mixed plantings can increase the decomposition rate of senescent leaves and rapidly release nutrients into the topsoil through microenvironmental changes such as reduced soil temperature, increased soil moisture, and improved microbial activity (Wang et al., 2014; Paula et al., 2015; Lin et al., 2022). Therefore, the SLNC and SLPC did not differ between the RPAD and RP stands but were lower in the RPAS stand than in the RP stand for *R. pseudoacacia* (**Figures 1c, d**; **Supplementary Table S4**), whereas the soil total N and P concentrations were greater in the mixed plantations than in the monocultures (**Supplementary Table S3**). Additionally, senescent leaves with high nutrient concentrations can enhance soil nutrient availability by altering the amount of belowground N and P inputs (Forrester et al., 2006; Voigtlaender et al., 2012; Ma et al., 2017; Waithaisong et al., 2020). Thus, the SLNC and SLPC of *A. davidiana* and *A. sibirica* were greater in mixed plantations than in monocultures (**Figures 1c, d**), suggesting that senescent leaves of these mixed species than the monocultures returned more nutrients into the soil. However, whether the amounts of P released by the high-quality senescent leaves of *A. davidiana* and *A. sibirica* were much greater than that consumed by rhizobia from *R. pseudoacacia* is still unclear. First, mixed species growth under water stress conditions may not compete for soil available-P because of the strongly reduced mobility of soil P (Lambers et al., 2008). Second, tree structure can affect the nutrient concentration of senescent leaves in plants (Hayes et al., 2014; Lin et al., 2022). For example, the SLNC and SLPC of *A. davidiana* and *A. sibirica* in monocultures and mixed stands were positively associated with the average tree height and crown area (**Figures 4b, c**). It follows that more N and P elements were released from senescent leaves and transported into two shorter, non-N_2_-fixing species.

### 4.2 Leaf nutrient resorption efficiency and its influencing factors

In general, the process of N fixation by *R. pseudoacacia* rhizobacteria could increase soil available N concentration (Nygren and Leblanc, 2015). However, the difference in *R. pseudoacacia* NRE between pure and mixed stands was not significant (**Figure 2a**), inconsistent with Hypothesis 2. Thus, the mixed planted *R. pseudoacacia* was not changed its demand for N. However, the PRE of *R. pseudoacacia* was lower in the RP stand than in the RPAD stand, whereas it was not significantly different between the RP and RPAS stands (**Figure 2b**). A possible explanation is that: 1) less P consumed during the N fixation than the soil-available P that absorbed by *R. pseudoacacia*, 2) senescent leaves were released enough P for *R. pseudoacacia*, and 2) microclimate could improved the process (Aerts, 1996; Paula et al., 2015; Sardans et al., 2021). For this reason, the decomposition of senescent leaves may release more P to the soil in the RPAS stand than in the RPAD stand. However, the NRE and PRE of *A. davidiana* and *A. sibirica* were lower in mixed stands than in monocultures (**Figure 2**), consistent with Hypothesis 2. These findings suggest that the growth of *A. davidiana* and *A. sibirica* in the mixed plantation was less limited by soil N and P than that in the monoculture. Thus, mixed *A. davidiana* and *A. sibirica* tend to fully utilize N fixed by rhizobia and reduce NRE (Augusto et al., 2013; Paula et al., 2015; Bu et al., 2019) and therefore better adapted to nutrient-poor soils (Brant and Chen, 2015; Sardans et al., 2017). Additionally, the NRE and PRE of *A. davidiana* and *A. sibirica* were negatively correlated with crown area (**Figures 4b, c**), suggesting that the N and P in stems and branches were mainly obtained from the soil but less from reabsorbed nutrients by senescent leaves. Furthermore, the NRE of *A. davidiana* was negatively correlated with the soil total N concentration (**Figure 4b**), and the PRE of *A. sibirica* was negatively correlated with the soil total P concentration (**Figure 4c**). Therefore, in mixed plantations, *A. davidiana* releases N to the soil through senescent leaves, whereas *A. sibirica* releases P.

Consistent with Hypothesis 2, the relative nutrient rsorption efficiency (RRE) of *R. pseudoacacia* in pure and mixed plantations was < 100% (**Figure 3a**; **Supplementary Table S4**), which agree with the greater capacity of *R. pseudoacaia* to uptake and retain P during N fixation (Blank and Morgan, 2014; Liu et al., 2017). Thus, the growth of *R. pseudoacacia* was mostly limited by soil P (Reed et al., 2012; Han et al., 2013; Cao and Chen, 2017). Particularly, the growth of *R. pseudoacacia* was more easily limited by soil P in the RPAD stand than in the RP stand (**Figure 3a**; **Supplementary Table S4**). However, inconsistent with Hypothesis 2, *A. davidiana* and *A. sibirica* in the pure plantations presented values of RRE > 100%, which were not significantly different between the pure and mixed plantations (**Figure 3a**). These findings suggest that mixed planting of *R. pseudoacacia* did not significantly alleviate the N limitation imposed on *A. davidiana* or *A. sibirica*. This may be related to the fact that the availability of elemental N has a critical effect on the growth and photosynthesis of plants (Elser et al., 2010), especially the higher N requirements of *A. davidiana* and *A. sibirica*, which have larger crown areas in mixed plantations than in monocultures (**Supplementary Table S2**).

The NRE of individuals and all species were positively correlated with PRE (**Figure 3b**), consistent with previous studies (Han et al., 2013; Deng et al., 2019). Particularly, *A. sibirica* NRE and PRE were significantly positively correlated (**Figure 3b**), indicating the positive regulation of *A. sibirica* NuRE in pure and mixed plantations by soil N and P limitation. Thus, the N and P distributed in the green/senescent leaves of *A. sibirica* were derived mainly from the soil. However, the nutrient sources for the green leaves of *R. pseudoacacia* was also influenced by the rhizobial N fixation (Aerts, 1996; Forrester et al., 2006; Paula et al., 2015). Specifically, rhizobia during N fixation could consume large amounts of soil P, resulting in the differences between soil available N and P concentrations in the rhizosphere (Nygren and Leblanc, 2015; Yuan et al., 2016; Sardans et al., 2021). Thus, NRE and PRE were not significantly positively correlated for *R. pseudoacacia* in pure and mixed plantations (**Figure 3b**). However, NRE and PRE were significantly positively associated for all species (**Figure 3b**). Therefore, tree mixing is conducive to coordinating the relationship between NRE and PRE for different species through soil N and P availability, although its effect on N_2_- and non-N_2_-fixing species shows individual variation (Forrester et al., 2006; Coll et al., 2018).

### 4.3 Relationships between leaf nutrient concentrations and nutrient resorption efficiencies

Consistent with Hypothesis 3, the negative correlation between NRE and the GLNC of different plantations were not significant for *R. pseudoacacia*, but they were significant for *A. davidiana* and *A. sibirica* (**Figure 7a**). These findings suggest that rhizobial activity can significantly affect the NRE of *R. pseudoacacia* but not those of *A. davidiana* and *A. sibirica.* The reason for this may be that the mixed *R. pseudoacacia* maintains NRE by enhancing rhizobial N fixation and fully utilizes the fixed N, allowing *A. davidiana* and *A. sibirica* to benefit from the N fixation of *R. pseudoacacia*, which does not compete with the other plants for soil available N (Augusto et al., 2013; Bu et al., 2019; Chen and Chen, 2022). Additionally, the GLNC of mixed *A. davidiana* and *A. sibirica* can also originate from other sources, such as N deposition, available N and minerals. However, soil P is the main resource of the GLPC for different species (Blank and Morgan, 2014); thus, soil-available P is the main nutrient limiting plant growth, whether it is an N_2_-fixing species or not. Thus, the PRE and GLPC were positively associated in *R. pseudoacacia* but negatively associated in *A. davidiana* and all species (**Figure 7b**), indicating a difference in P uptake strategies between *R. pseudoacacia* and *A. davidiana* and *A. sibirica*. Sardans et al. (2012), Liu et al. (2017), and Li et al. (2018) reported that plant strategies for nutrient uptake were based on energy allocation to leaves and roots, with plants preferring to absorb more nutrients at a lower energy cost. On this basis, the energy cost of the green leaves of *R. pseudoacacia* was lower to retain P to compensate for soil P deficiency, whereas the energy cost of the roots of *A. davidiana* was lower during root nutrient uptake.

According to the theory of nutrient uplift, plants growth were mostly dependented on nutrients in the topsoil (Jobbagy and Jackson, 2001), and therefore the high nutrient concentrations in senescent leaves were associated with low nutrient resorption efficiency but high soil nutrient concentration (Aerts and Chapin, 1999; Sardans et al., 2017). Thus, the NRE of individual species was significantly negatively correlated with SLNC (**Figure 7c**), which is consistent with Hypothesis 3 and suggests an inverse control of SLNC on NRE. Furthermore, plants growing under high soil total N concentrations tend to withdraw a lower percentage of N from their leaves, resulted in more N returning to the topsoil via SLNC (Jobbagy and Jackson, 2001; Hayes et al., 2014; Brant and Chen, 2015). However, the negative relationship between PRE and SLPC was not significant for *R. pseudoacacia*, but the relationships were significant for *A. davidiana* and *A. sibirica* (**Figure 7d**), consistent with Hypothesis 3. These results indicate that other factors influence the process of P supply to *R. pseudoacacia*, such as P consumption by rhizobia during N fixation; however, tree mixture has a weak effect on P cycling in *A. davidiana* and *A. sibirica* (Sardans et al., 2012; Cierjacks et al., 2013). Therefore, the decomposition of senescent leaves can provide sufficient N for plant growth, but the P concentration does not meet the requirements of *R. pseudoacacia*, although it is sufficient for *A. davidiana* and *A. sibirica* (Jobbagy and Jackson, 2001; Ma et al., 2017).

Over, the results demonstrated that *A. davidiana* and *A. sibirica* are suitable mixing species to be planted with *R. pseudoacacia* to improve N and P cycling. In future forest management and ecosystem restoration planning, appropriate mixed species compositions and their effects on stand characteristics should be the first considerations to maintain the nutrient cycling of temperate forests.

## 5 Conclusions

In the Loess Hilly Region, *Robinia pseudoacacia* is often mixed with *Amygdalus davidiana* and *Armeniaca sibirica* to increase the ecological and economic benefits. However, how these plant mixtures affect the nutrient status of the individual species remains uncertain. The results revealed the variations in leaf N and P concentrations and resorption efficiencies between monocultures and mixed species and their relationships with influencing factors. The main conclusions are as follows:

1. The GLNC of *R. pseudoacacia* in mixed stands was influenced by specific leaf area and soil total N cocentration, whereas that of *A. davidiana* and *A. sibirica* more by soil total N concentration than specific leaf area. The GLPC of *R. pseudoacacia* in mixed plantations was influenced by the soil sand content and total P concentration; however, the higher GLPC in mixed plantations than in monocultures for *A. davidiana* and *A. sibirica* was influenced by the crown area, soil sand content, and senescent leaves. The SLNC and SLPC of *A. davidiana* and *A. sibirica* in monocultures and mixed stands were positively associated with the average tree height and crown area, suggests that amounts of N and P released from senescent leaves and transported into the non-N_2_-fixing species.
2. Mixed planting led to the variation in the PRE of *R. pseudoacacia* but decreased the NRE and PRE of *A. davidiana* and *A. sibirica*, because mixed *R. pseudoacacia* utilized more P during the N fixation, and the decomposition of senescent leaves supplied enough nutrients for *A. davidiana* and *A. sibirica*. Mixed planting did not significantly alleviated P limitation for *R. pseudoacacia* or N limitation for *A. davidiana* and *A. sibirica* because the N fixation process consumes P, and the greater crown areas of *A. davidiana* and *A. sibirica* require more N in mixed plantations than in monocultures. Regardless of the individual differences in the effects of the tree mixture on N_2_- and non-N_2_-fixing species, the coordinated association between NRE and PRE was still enhanced by the soil N and P concentrations.
3. In general, *R. pseudoacacia* prefers to reabsorb P by green leaves, whereas *A. davidiana* uptake nutrient to compensate for soil P deficiency. Rhizobacterial activity can significantly affect the NRE of *R. pseudoacacia* but not those of *A. davidiana* and *A. sibirica*. Decomposition of senescent leaves provides sufficient N and insufficient P for *R. pseudoacacia* and sufficient N and P for *A. davidiana* and *A. sibirica*.

## Data Availability

The original contributions presented in the study are included in the article/**Supplementary Material**. Further inquiries can be directed to the corresponding authors.
