# Supplementary material for: Impact of mixed plantation strategies on the nutrient concentrations of green and senescent leaves and their nutrient resorption efficiencies in temperate forests of the Loess Hilly Region
Source: Front Plant Sci. 2025 May 13;16:1527930. doi: 10.3389/fpls.2025.1527930 (PMC12118354; doi:10.3389/fpls.2025.1527930)
Supplement: Supplementary file 1 [file Table1.docx]

**Table S1** Information of sampling sites.

| Stand types | Stands | No. | Latitude (N) | Longitude (E) | Altitude (m) | Gradient (°) |
| --- | --- | --- | --- | --- | --- | --- |
| Monocultures | RP | 1 | 36°35′51″ | 109°13′37″ | 1203 | 21.4 |
|  | RP | 2 | 36°35′51″ | 109°13′40″ | 1200 | 21.7 |
|  | RP | 3 | 36°35′25″ | 109°13′56″ | 1197 | 14.0 |
|  | AD | 4 | 36°35′30″ | 109°13′20″ | 1217 | 28.8 |
|  | AD | 5 | 36°35′50″ | 109°13′37″ | 1202 | 25.0 |
|  | AD | 6 | 36°35′47″ | 109°13′39″ | 1201 | 21.0 |
|  | AS | 7 | 36°35′49″ | 109°13′43″ | 1160 | 24.0 |
|  | AS | 8 | 36°35′49″ | 109°13′57″ | 1222 | 18.6 |
|  | AS | 9 | 36°36′40″ | 109°16′32″ | 1224 | 17.3 |
| Mixed stands | RPAD | 10 | 36°35′52″ | 109°13′43″ | 1196 | 20.4 |
|  | RPAD | 11 | 36°35′29″ | 109°13′27″ | 1191 | 29.2 |
|  | RPAD | 12 | 36°35′30″ | 109°13′12″ | 1199 | 22.6 |
|  | RPAS | 13 | 36°35′34″ | 109°16′7″ | 1192 | 22.3 |
|  | RPAS | 14 | 36°35′21″ | 109°13′51″ | 1191 | 23.9 |
|  | RPAS | 15 | 36°35′34″ | 109°13′45″ | 1203 | 20.4 |

**Note:** RPAD, *R. pseudoacacia* and *A. davidiana* mixed stand; RPAS, *R. pseudoacacia* and *A. sibirica* mixed stand; RP, *R. pseudoacacia* monoculture; AD, *A. davidiana* monoculture; AS, *A. sibirica* monoculture.
