# Supplementary material for: Impact of mixed plantation strategies on the nutrient concentrations of green and senescent leaves and their nutrient resorption efficiencies in temperate forests of the Loess Hilly Region
Source: Front Plant Sci. 2025 May 13;16:1527930. doi: 10.3389/fpls.2025.1527930 (PMC12118354; doi:10.3389/fpls.2025.1527930)
Supplement: Supplementary file 2 [file Table2.docx]

**Table S2** Stand characteristics of three different tree species in pure and mixed stands.

| Stands | Tree species | Tree structures |  |  |  | Leaf functional traits | | |
| --- | --- | --- | --- | --- | --- | --- | --- | --- |
|  |  | Average tree  height (m) | Diameter breast height (cm) | Crown area  (m^2^) |  | Specific leaf area  (cm^2^ g^-1^)  [Equation 1] | Leaf dry matter  content (g g^-1^)  [Equation 2] | Leaf tissue density  (g cm^-3^)  [Equation 3] |
| RP | *R.pseudoacacia* | 9.54±0.41a | 11.55±0.96a | 6.52±0.50b |  | 335.17±5.91a | 0.28±0.01c | 0.32±0.01c |
| RPAD |  | 8.52±0.22b | 9.30±0.22b | 4.71±0.38c |  | 251.59±7.68bc | 0.33±0.01c | 0.37±0.02c |
| RPAS |  | 8.44±0.18b | 9.21±0.24b | 6.24±0.35b |  | 259.04±6.67b | 0.32±0.00c | 0.38±0.00c |
| AD | *A. davidiana* | 3.58±0.12d | 6.32±0.12c | 4.88±0.05c |  | 114.83±6.37e | 0.48±0.03a | 0.53±0.02a |
| RPAD |  | 4.11±0.05d | 5.16±0.15c | 6.19±0.08b |  | 216.27±12.14cd | 0.30±0.00c | 0.32±0.01c |
| AS | *A. sibirica* | 3.46±0.05d | 10.09±0.77ab | 4.26±0.21c |  | 109.85±2.98e | 0.39±0.00b | 0.43±0.02 b |
| RPAS |  | 5.62±0.12c | 9.75±0.12b | 7.42±0.07a |  | 187.62±9.71d | 0.33±0.02c | 0.32±0.01c |
| One-way | F | 166.82 | 21.0987 | 15.93 |  | 106.98 | 27.64 | 29.66 |
| ANOVA | *P* | *** | *** | *** |  | *** | *** | *** |

**Note:** RPAD, *R. pseudoacacia* and *A. davidiana* mixed stand; RPAS, *R. pseudoacacia* and *A. sibirica* mixed stand; RP, *R. pseudoacacia* monoculture; AD, *A. davidiana* monoculture; AS, *A. sibirica* monoculture. Different lowercase letters represent significant differences among the same/different species for the same/different stands (***, *P* < 0.001).
