# Supplementary material for: Impact of mixed plantation strategies on the nutrient concentrations of green and senescent leaves and their nutrient resorption efficiencies in temperate forests of the Loess Hilly Region
Source: Front Plant Sci. 2025 May 13;16:1527930. doi: 10.3389/fpls.2025.1527930 (PMC12118354; doi:10.3389/fpls.2025.1527930)
Supplement: Supplementary file 3 [file Table3.docx]

**Table S3** Soil physicochemical properties of monoculture and mixed stands.

| Stand types | Stands | Soil physical properties | | | | |  | Soil chemical properties | | | | |
| --- | --- | --- | --- | --- | --- | --- | --- | --- | --- | --- | --- | --- |
|  |  | Soil pH | Bulk  density  (g cm^-3^) | Sand  Content  (%) | Silt  content  (%) | Silt  content  (%) |  | Organic carbon concentration  (g kg^-1^) | Total nitrogen concentration(g kg^-1^) | Total phosphorus concentration  (g kg^-1^) | Available nitrogen concentration(mg kg^-1^) | Available phosphorus concentration  (mg kg^-1^) |
| Monoculture | RP | 8.80±0.01 | 1.48±0.07 | 14.79±0.56c | 55.05±1.10a | 30.16±0.57b |  | 5.00±0.40 | 0.51±0.03 | 0.57±0.00b | 12.02±0.17 | 2.30±0.17ab |
|  | AD | 8.85±0.06 | 1.49±0.01 | 15.66±0.12bc | 54.07±0.35ab | 30.28±0.32b |  | 3.97±0.41 | 0.41±0.04 | 0.58±0.01ab | 11.39±0.97 | 1.28±0.15c |
|  | AS | 8.88±0.03 | 1.52±0.07 | 17.69±0.49a | 48.40±0.67c | 33.91±0.19a |  | 5.45±1.00 | 0.47±0.08 | 0.53±0.02c | 10.23±1.14 | 1.63±0.11bc |
| Mixed stand | RPAD | 8.64±0.16 | 1.51±0.03 | 16.73±0.43ab | 51.86±1.37b | 31.40±1.06b |  | 5.80±0.96 | 0.57±0.08 | 0.60±0.01ab | 12.44±0.45 | 2.41±0.42a |
|  | RPAS | 8.66±0.04 | 1.53±0.04 | 15.17±0.18c | 54.46±0.71ab | 30.37±0.54b |  | 5.65±0.55 | 0.57±0.05 | 0.61±0.00a | 12.63±0.25 | 1.93±0.11abc |
| One-way | F | 1.98 | 0.19 | 9.08 | 8.93 | 6.65 |  | 1.06 | 1.29 | 14.12 | 1.85 | 4.42 |
| ANOVA | *P* | ns | ns | ** | ** | ** |  | ns | ns | *** | ns | * |

**Note:** RPAD, *R. pseudoacacia* and *A. davidiana* mixed stand; RPAS, *R. pseudoacacia* and *A. sibirica* mixed stand; RP, *R. pseudoacacia* monoculture; AD, *A. davidiana* monoculture; AS, *A. sibirica* monoculture. Different lowercase letters represent significant differences among different stands for the same/different stand types (***, *P* < 0.001; **, *P* < 0.01; *, *P* < 0.05; ns, *P* > 0.05).
