# Supplementary material for: Impact of mixed plantation strategies on the nutrient concentrations of green and senescent leaves and their nutrient resorption efficiencies in temperate forests of the Loess Hilly Region
Source: Front Plant Sci. 2025 May 13;16:1527930. doi: 10.3389/fpls.2025.1527930 (PMC12118354; doi:10.3389/fpls.2025.1527930)
Supplement: Supplementary file 4 [file Table4.docx]

**Table S4** The percentage changes in green leaf nitrogen (ΔGLNC) and phosphorus (ΔGLPC) concentrations, senescent leaf nitrogen (ΔSLNC) and phosphorus (ΔSLPC) concentrations, and nitrogen (ΔNRE) and phosphorus (ΔPRE) resorption efficiencies of different tree species between mixed stands and monocultures.

| Tree species | Mixture-Pure | ΔGLNC (%)  [Equation 4] | ΔGLPC (%)  [Equation 4] | ΔSLNC (%)  [Equation 5] | ΔSLPC (%)  [Equation 5] | ΔNRE (%)  [Equation 9] | ΔPRE (%)  [Equation 9] |
| --- | --- | --- | --- | --- | --- | --- | --- |
| *R. pseudoacacia* | RPAD-RP | 0.21% | 15.70% | 3.06% | -0.33% | -2.76% | 10.73% |
| *R. pseudoacacia* | RPAS-RP | -6.41% | -1.65% | -14.44% | -13.33% | 8.47% | 7.80% |
| *A. davidiana* | RPAD-AD | 44.48% | 35.23% | 83.08% | 66.67% | -29.93% | -29.35% |
| *A. sibirica* | RPAS-AS | 28.83% | 14.29% | 42.75% | 22.41% | -12.21% | -9.31% |

**Note:** RPAD, *R. pseudoacacia* and *A. davidiana* mixed stand; RPAS, *R. pseudoacacia* and *A. sibirica* mixed stand; RP, *R. pseudoacacia* monoculture; AD, *A. davidiana* monoculture; AS, *A. sibirica* monoculture.
